# Suoquan pill for the treatment of diabetic nephropathy: A protocol for systematic review and meta-analysis

**DOI:** 10.1097/MD.0000000000025613

**Published:** 2021-04-30

**Authors:** Piao Cai, Zhu Wu, Wei Huang, Qian Niu, Ye Zhu, Dehui Yin

**Affiliations:** a College of Basic Medicine, Chengdu University of Traditional Chinese Medicine, Chengdu, Sichuan; b The First Affiliated Hospital of Hainan Medical University, Haikou, Hainan; c College of TCM of Hainan Medical University, Haikou, Hainan, China.

**Keywords:** diabetic nephropathy, protocol, randomized controlled trial, suoquan pill, systematic evaluation, traditional Chinese medicine

## Abstract

**Background:**

Diabetic nephropathy (DN) is one of the most common complications of diabetes and the main cause of kidney failure in developed countries. Clinically, DN is usually treated by controlling blood sugar and blood pressure. According to reports, the application of angiotensin-converting enzyme inhibitor (ACEI) or angiotensin receptor antagonist can only target a certain stage of disease development. However, the application of Suoquan Pill (SQP) in traditional Chinese medicine has produced obvious clinical effects and minor side effects. It is used to treat DN and other diseases, but there is no systematic review of SQP in the treatment of diabetic nephropathy. This article reviews the effectiveness and safety of SQP in the treatment of DN.

**Methods:**

The database sets the registration date for randomized controlled trials (RCT) to March 25, 2021. By searching the following eight Chinese and English electronic databases: Cochrane Library, Embase, PubMed, Science Net, China National Knowledge Infrastructure, China Biomedical Literature Database. Chinese scientific journal database and Wanfang database for analysis. The main results are clinical efficacy, urinary albumin excretion rate, symptom score and quality of life. Finally, Stata 15 was used for meta-analysis.

**Results:**

This study will provide the latest evidence for SQP in the treatment of DN in the following aspects: clinical efficacy, urinary albumin excretion rate, quality of life, symptom score.

**Conclusion:**

The results of this study will provide evidence for evaluating the effectiveness of SQP in the treatment of DN.

**OSF registration number:**

10.17605/OSF.IO/KZ9RA

## 1 Introduction

As one of the most important complications of diabetes, the morbidity and mortality of diabetic nephropathy (DN) are increasing rapidly in the world population.^[1,2]^ DN is pathologically characterized by glomerular hypertrophy, mesangial matrix enlargement, and even glomerular fibrosis and sclerosis. Microalbuminuria is the main symptom in the early clinical stage. Progressive renal damage, hypertension, and edema may occur in further development. In severe cases, renal failure may occur.^[3]^ Among them, elevated UAE is an important indicator of early diagnosis of DN. Urinary albumin excretion rate (UAE) is also related to the onset of diabetic complications including hypertension, hyperlipidemia, atherosclerosis, and cardiovascular disease.^[4]^ Angiotensin converting enzyme inhibitor (ACEI) has pharmacological effects such as improving renal hemodynamics, reducing urine protein excretion, inhibiting the activity of mesangial cells, fibroblasts, and macrophages, and improving membrane permeability. Even if the systemic blood pressure is normal, it can also produce renal protection. However, ACEI has the risk of hyperkalemia, renal dysfunction and dry cough in the treatment of DN.^[5]^ The development and application of traditional Chinese medicine has opened up a new milestone for the treatment of DN. A large number of clinical studies have shown that Chinese medicine plays a key role in the treatment of ND.^[6]^

Suoquan Pill (SQP) is derived from the “Prescriptions for Women” written by Xue Ji in the Ming Dynasty. It mainly treats kidney deficiency, frequent urination, and enuresis. It can be used to treat DN with obvious curative effect.^[7]^ The product is mainly composed of Yizhiren (*Alpinia oxyphylla*) and Wuyao (*Lindera root*) groups. The two medicines of Yizhiren and Wuyao can be used in combination to warm and nourish the spleen, dispel cold pathogens, replenish the spleen and kidney, and reduce frequent urination. Modern studies have found that Yizhiren can reduce the proteinuria level of experimental animals, regulate the renin-angiotensin-aldosterone system (RAAS), inhibit oxidative stress, reduce the proliferation of glomerular mesangial cells, regulate the detrusor of the bladder, and down-regulate the cycle The protein kinase inhibitor P27 (p27kip1) protein can improve renal function, protect kidney morphology, and delay renal failure to a certain extent.^[8–11]^ Wuyao has anti-inflammatory and analgesic, antioxidant, anti-fatigue, anti-viral, antibacterial, pharmacological effects such as regulating the digestive tract, relaxing visceral smooth muscle, and improving the function of the central nervous system.^[12]^ A large number of recent clinical studies have shown that SQP has a significant effect on the treatment of DN.^[13,14]^ There is an urgent need for a systematic review to support the effectiveness and safety of SQP in the treatment of DN. Therefore, the purpose of this study is to systematically review the existing literature to evaluate the effectiveness and safety of SQP in the treatment of DN patients.

## 2 Methods

### 2.1 Protocol and registration

The scheme was formulated according to the preferred reporting items of the Guidelines for Systematic Review and Meta-Analysis (PRISMA-P). It has been registered on the Open Science Framework (OSF) platform (https://osf.io/azkuq), the registration number: 10.17605/OSF.IO/KZ9RA

### 2.2 Types of studies

Randomized controlled clinical trials (RCT) published in English and Chinese will be selected, whether blind or not. Without time constraints.

### 2.3 Types of patients

This review will consider all relevant studies SQP in the treatment of DN. There are no restrictions of gender, race, or age.

### 2.4 Types of interventions

The intervention method of the experimental group is SQP. There are no restrictions on administration time, frequency, and dosage form. Research combined with other interventions (such as acupuncture) will be excluded.

### 2.5 Outcomes

The following main outcomes will be measured: clinical efficiency, UAE reduction rate, quality of life, and symptom scores.

### 2.6 Search strategy

#### 2.6.1 Electronic searches

From the establishment of each database to March 25, 2021 2, a comprehensive search will be conducted through the following electronic databases: Cochrane Library, Embase, PubMed, Web of Science, Chinese Scientific Journal Database, China National Knowledge Infrastructure, China Biomedical Literature Database, and Wanfang Database. Key words include “Suoquan Pill,” “ diabetic nephropathy,” etc. The initial search methods of PubMed are shown in Table 1. Relevant data will also be searched through other sources.

**Table 1:** Search strategy of the PubMed.

Number	Search terms
#1	Diabetic nephropathy [Mesh]
#2	Diabetes [Title/Abstract] kidney disease [Title/Abstract]
#3	#1 OR #2
#4	Suoquan Pill[Title/Abstract]
#5	Suoquan wan[Title/Abstract]
#6	#4 AND #5
#7	randomized controlled trial[Publication Type]
#8	controlled clinical trial[Publication Type]
#9	randomized[Title/Abstract]
#10	randomly[Title/Abstract]
#11	#10 OR #11 OR #12 OR #13
#12	#3 AND #6 AND #11

### 2.7 Data collection and analysis

#### 2.7.1 Selection of studies

**Figure 1:**
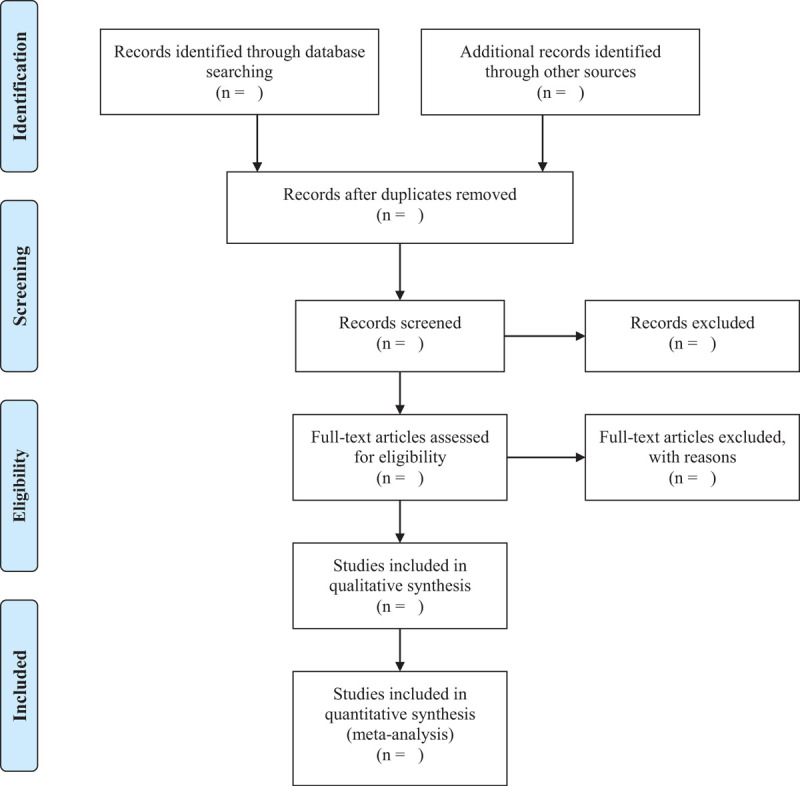
PRISMA flow diagram of the study selection process.

As shown in Figure 1, two researchers (Piao Cai and Zhu Wu) imported the retrieved documents into Endnote X9 software for review, deleted duplicate references, and initially screened the abstract to exclude documents that obviously did not meet the inclusion criteria. Then, Download and read the full text for follow-up inspection. After the final inclusion, we will use the pre-designed data extraction table to extract data and cross-check the results. If there is any objection, we will ask a third researcher (Wei Huang) to assist in making a judgment.

#### 2.7.2 Data extraction

Data extraction will be conducted by two researchers (Qian Niu and Ye Zhu) in a standardized abstract form of data, consisting of four parts: basic information of the study, characteristics of the test subjects, intervention measures, research results and measurement data. Similarly, if there is any discrepancy, the final decision will be made by the third reviewer (Dehui Yin). If there is missing or insufficient information, we will analyze and deal with it, and consider the impact of missing data on the meta-analysis results.

#### 2.7.3 Quality evaluation on methodology

According to the Cochrane system—ventions v. 5.2.0 system evaluation handbook (update) in June 2017 standards, including seven domains: random distribution method is hidden and blind patients, dazzling the results of the assessment, incomplete data processing results (i.e., whether to describe the subsequent losses, the number of the export, whether intentionally analysis), selective reports, other bias. The quality of the included literature will be divided independently by 2 researchers into 3 categories (low risk, high risk, and unclear). If there is disagreement, consensus will be reached through discussion or consultation with the third author.

#### 2.7.4 Statistic analysis

Meta-analysis was performed using Stata 15. The results of the dichotomy will be expressed as the relative risk (RR) or odds ratio (OR) of the 95% confidence interval (CI). For continuous outcomes, if the outcome measurements of alzl studies are based on the same measurement unit, then the mean difference (MD) of 95% CI is given; otherwise, the standard mean difference (SMD) of 95% CI is given for analysis.

#### 2.7.5 Assessment of heterogeneity

Statistical heterogeneity between studies will be assessed by I2 and chi-square statistics. If I2 is between 50% and 100%, there is a statistical heterogeneity, for which we will use a random effects model to analyze the data. If the heterogeneity test is not significant (*I*^2^ ≤ 50%), the fixed-effect model is used. In addition, due to differences in heterogeneity, we will conduct subgroup or sensitivity analysis to look for potential causes.

#### 2.7.6 Assessment of reporting bias

Funnel plots will be drawn to assess report bias. If a potential reporting basis is found, the Berg and Egger tests will be used to assess funnel plots for symmetry and perceived publication bias.

#### 2.7.7 Sensitivity analysis

Sensitivity analysis was used to evaluate the robustness of the main efficacy indicators. The method was to eliminate the low-quality studies one by one, merge the data, and evaluate the impact of sample size, research quality, statistical methods and missing data on the meta-analysis results.

#### 2.7.8 Subgroup analysis

If the results are heterogeneous, we will conduct a subgroup analysis based on different reasons. Heterogeneity is mainly manifested in race, age, gender, drug dosage form, different forms of intervention, course of treatment, doseand other aspects.

#### 2.7.9 Quality of evidence

The reliability of the evidence will be assessed by grading recommendations for evaluation, development, and evaluation. The quality of evidence will be classified as high, medium, low, or very low.

#### 2.7.10 Ethics and dissemination

Our goal is to publish this review in a peer-reviewed journal. Private information from individuals is not subject to review and therefore no informed consent is required. Ethical approval is also unnecessary because the study is not a clinical trial. Patients and the public did not participate.

## 3 Discussions

DN is a common complication of diabetes, and DN has become the main cause of renal failure in developed countries.^[15]^ The pathogenesis of DN is relatively complex, involving various factors such as glucose and lipid metabolism disorder, abnormal hemodynamics, oxidative stress, participation of multiple cytokines, and heredity. Its pathogenesis is slow and takes a long time, requiring long-term treatment. Although various clinical treatments for DN have reduced the risk of its deterioration, DN is still the primary cause of end-stage renal disease (ESRD).^[16,17]^ In addition to the health problems of patients, DN also brings a heavy burden to society. At present, the treatment of angiotensin converting enzyme inhibitor (ACEI) is used to reduce urine protein, which has a single effect and obvious side effects.^[18]^ The advantages of traditional Chinese medicine in the treatment of DN are increasingly apparent, not only can alleviate the suffering of patients, but also can fundamentally solve the suffering of patients. The cause of the disease is to grasp the pathogenesis to regulate the patient's physique, enhance the body's transport, chemical action and transport function. Modern pharmacological studies have also confirmed that SQP has a clear pharmacological effect on DN, with multi-component, multi-target, and multi-step therapeutic effects. However, a systematic review of SQP treatment of DN has not been published. This systematic review will be the first to summarize the current state of the evidence on the effectiveness and safety of SQP in the treatment of DN. This assessment will help doctors and patients with DN.

## Author contributions

Piao Cai and Zhu Wu made similar contributions to literature retrieval and research, and wrote the first draft of the agreement. Qian Niu developed a search strategy. Piao Cai and Zhu Wu will conduct literature retrieval and collation. Wei Huang, Ye Zhu and Dehui Yin will assess the risk of bias in the literature. Data analysis and article writing will be completed by Piao Cai and Qian Niu. As corresponding author, Dehui Yin and Ye Zhu will be responsible for supervising every process of audit and controlling the quality of research. All the authors approved the publication of the program.

**Conceptualization:** Qian Niu.

**Data curation:** Piao Cai, Qian Niu, Ye Zhu.

**Formal analysis:** Wei Huang, Qian Niu.

**Funding acquisition:** Qian Niu, Ye Zhu, Dehui Yin.

**Investigation:** Piao Cai, Zhu Wu.

**Project administration:** Ye Zhu, Dehui Yin.

**Resources:** Dehui Yin.

**Supervision:** Wei Huang, Dehui Yin.

**Validation:** Dehui Yin.

**Writing – original draft:** Piao Cai, Zhu Wu.

**Writing – review & editing:** Piao Cai, Dehui Yin.
